# Explainable protein function annotation using local structure embeddings

**DOI:** 10.1101/2023.10.13.562298

**Published:** 2023-10-16

**Authors:** Alexander Derry, Russ B. Altman

**Affiliations:** 1 Department of Biomedical Data Science, Stanford University, Stanford, CA; 2 Departments of Bioengineering, Genetics, and Medicine, Stanford University, Stanford, CA

## Abstract

The rapid expansion of protein sequence and structure databases has resulted in a significant number of proteins with ambiguous or unknown function. While advances in machine learning techniques hold great potential to fill this annotation gap, current methods for function prediction are unable to associate global function reliably to the specific residues responsible for that function. We address this issue by introducing PARSE (Protein Annotation by Residue-Specific Enrichment), a knowledge-based method which combines pre-trained embeddings of local structural environments with traditional statistical techniques to identify enriched functions with residue-level explainability. For the task of predicting the catalytic function of enzymes, PARSE achieves comparable or superior global performance to state-of-the-art machine learning methods (F1 score > 85%) while simultaneously annotating the specific residues involved in each function with much greater precision. Since it does not require supervised training, our method can make one-shot predictions for very rare functions and is not limited to a particular type of functional label (e.g. Enzyme Commission numbers or Gene Ontology codes). Finally, we leverage the AlphaFold Structure Database to perform functional annotation at a proteome scale. By applying PARSE to the dark proteome—predicted structures which cannot be classified into known structural families—we predict several novel bacterial metalloproteases. Each of these proteins shares a strongly conserved catalytic site despite highly divergent sequences and global folds, illustrating the value of local structure representations for new function discovery.

## Introduction

Proteins are complex molecules that perform a diverse range of biochemical functions, including molecular binding and transport, cellular signaling, and reaction catalysis. Identifying the set of functions performed by a protein is critical for elucidating its role in biological processes, which in turn enables greater understanding of disease pathogenesis and more precise targeting of therapeutics. Large-scale sequencing efforts and improvement in both experimental and computational techniques have resulted in the rapid expansion of sequence databases such as the Uniprot Knowledgebase (UniprotKB) (1), which has more than doubled in size in the last five years to around 250 million protein sequences. UniprotKB is the primary repository for annotations of protein function, including membership in protein family databases (e.g. Pfam (2), InterPro (3)) and classification to controlled terms from ontologies such as the Gene Ontology (GO) (4) or Enzyme Commission (EC) (5). However, experimental characterization or expert assessment of a protein’s function are infeasible at such scale, resulting in a significant annotation gap—the manually curated subset of UniprotKB (SwissProt (6)) contains less than 0.3% of the full database, and this proportion is rapidly shrinking.

In addition to global assignment of protein function, the identification of amino acids involved in each biochemical action is crucial for understanding a protein’s mechanism of action and to guide protein engineering and design efforts, which are often precisely targeted at specific functional sites. However, here the annotation disparity is even more stark: over 60% of proteins assigned an enzymatic function (i.e. EC number) in SwissProt have no active site residues identified. Curated databases of residue-level annotations are inherently limited in scope by the effort required to update and maintain them. For example, the Catalytic Site Atlas (CSA) (7), which contains detailed information about the residues involved in the enzyme catalytic mechanisms, is limited to one reference sequence and structure for each curated enzymatic function and is not being regularly updated.

The development of computational methods for predicting protein function is therefore a major challenge in protein science. Domain-specific profile hidden Markov models built on multiple alignments of homologous sequences (8–11) have traditionally been a dominant approach and form the basis of most protein family databases (2, 3, 12). To address the limitations of annotation transfer via homology, machine learning (ML) methods that integrate features from sequence, structure, and/or protein interaction networks have been developed for *de novo* function classification (13–19). Recent methods have leveraged self-supervised deep learning techniques such as protein language modeling (20–22), which can learn complex patterns from massive datasets without explicit feature engineering, to establish a new state of the art for protein function classification (23–27). However, while ML methods continue to improve, they have several limitations as general-purpose tools for function annotation.

First, to assemble sufficiently large labeled training datasets, many methods rely on pre-defined labels which are often broad or ambiguous. For example, GO terms have varying levels of specificity and single terms may overlap or contain multiple distinct biochemical functions (28). Similarly, although EC numbers are arranged in a four-level hierarchy with a more consistent level of specificity at the lowest level, some EC numbers are so rare that they are either excluded from training or aggregated up to a higher level of the tree. The imbalance in class sizes also results in decreased performance for rare function classes, further exacerbating the bias towards well-studied proteins (29). A recent method, CLEAN (30), improved performance on rare proteins by introducing a contrastive learning procedure. However, updating any supervised model with additional data or new labels requires re-training from scratch, adding overhead and potentially changing its performance characteristics.

Second, as sequence databases expand to species from across the tree of life (e.g. microbial metagenomes), it is important to be able to accurately annotate sequences that have low similarity to previously studied proteins. Methods which operate directly on protein structure provide a natural solution to this issue since structure is much more conserved than sequence and the biochemical activities of a protein in the cell are determined directly by its 3D conformation. However, the utility of such methods for function annotation has been limited by the availability of high-quality structure data, both in the context of training models (limited structures with functional labels) and of applying them at scale (most proteins of unknown function have only sequence available). Recent advances in structural biology have greatly increased the number of experimental structures in the Protein Data Bank (PDB) (31), allowing for large-scale function prediction models to be trained directly on 3D structure. For example, DeepFRI (24) combines a spatial graph of residues in the structure with sequence-based features from a protein language model. Additionally, the release of high-quality predicted structures for over hundreds of millions of proteins via AlphaFold (32, 33) and ESMFold (22) provide the opportunity to apply structure-based function prediction models to unannotated proteins at an unprecedented scale. These methods have already resulted in the discovery of an array of novel structural folds in need of annotation (34–36).

Third, methods for global prediction neglect the problem of residue-level annotation. As a result, local annotations are typically made using separate models built specifically for each functional site. These may be based on sequence motifs (37), manually defined rules (38, 39), or local structural representations (40–42), but they are all inherently limited by the need to individually develop a model for each function as well as a method for scanning over a protein of interest to discover potential hits (43). Some global ML methods, including DeepFRI and ProteInfer (27), attempt to identify key amino acids using class activation mapping (44), which uses the gradients of the trained model *post-hoc* to identify regions of the input which contribute to the prediction. However, these explainability techniques tend to be imprecise at a residue level, are not robust to spurious correlations (45, 46), and are typically evaluated qualitatively. Moreover, without reliable identification of functional residues the global predictions themselves may be misleading; for example, consider an enzymatic domain which is lacking a single catalytic residue critical to its function and is therefore inactive. Indeed, the lack of methods which can make accurate global predictions and provide residue-level explainability is cited as a major reason why few newly developed functional predictors are widely adopted by experimental biologists (28).

In light of these limitations, we present PARSE (Protein Annotation by Residue-Specific Enrichment), a knowledge-based method for automated function prediction which (1) predicts specific biochemical function and does not require large amounts of training data, with only one reference example required for each class; (2) leverages rich local structure representations pre-trained on evolutionary relationships across the PDB to capture functionally relevant features; and (3) simultaneously provides both a global function prediction and the individual residues which contribute to the prediction. Focusing on the task of enzymatic function prediction, we show that PARSE achieves comparable performance on global classification to current methods while greatly improving explainability by providing high-precision annotations of residues in the catalytic site. Using the AlphaFold Structure Database (AlphaFoldDB), we expand annotation coverage in the human proteome and provide novel functional hypotheses for several structural folds in the “dark proteome”, structures which do not share significant similarity to any annotated proteins. PARSE is open-source and available as a command-line Python tool at https://github.com/awfderry/PARSE.

## Results

### Identifying enriched functions by aggregating over site-level predictions.

**Figure 1.**
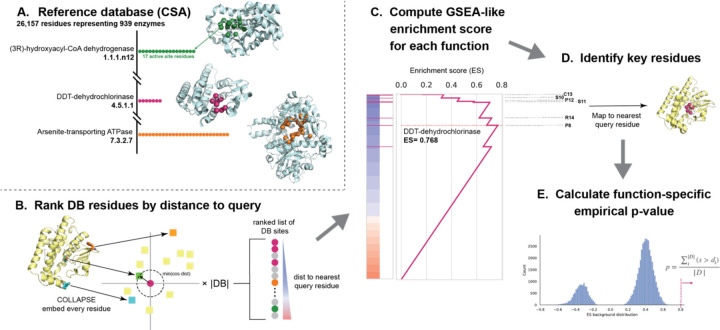
The PARSE algorithm for interpretable protein function annotation. Starting from top left, we first **(A)** build a reference database containing all residues associated with each functional group (here, enzymes from the Catalytic Site Atlas). Then, for a query protein to be annotated, we **(B)** embed the local environment around each residue using COLLAPSE (colored squares) and compute the pairwise cosine distance to the embedding of each residue in the reference database (colored circles). Database residues are then ranked by the minimum distance to any residue in the query and **(C)** an enrichment score is computed for each functional group relative to this ranked list. **(D)** Key residues for a given function are mapped to the query protein using the leading-edge subset of database residues which achieve scores greater than the maximum running enrichment score in the ranked list. Finally, to assess significance and reduce the influence of low-specificity functional labels, we **(E)** compute an empirical p-value based on a function-specific background score distribution.

In order to make explainable global predictions, we start by identifying sites with high similarity to a database of known functional residues before aggregating these site-level predictions to identify enriched functions at the protein level. This reflects the concept that a protein’s function depends on the presence of and interactions between several key functional residues and the structural environments surrounding them. Specifically, the PARSE algorithm consists of several key components (Fig. 1; Materials and Methods).

First, we define a database of protein structures annotated with a global functional class and the set of residues which contribute to that function. For enzymes we choose the Catalytic Site Atlas (CSA) (7), a curated database of experimentally-validated enzymatic functions with high-quality reference structures in the PDB. Then, for any input protein, we rank all residues in the reference database by their local structural similarity to any residue in the query. This requires a method for efficiently comparing the local structural environment surrounding individual residues. For this purpose, we use representations generated by COLLAPSE (42), a deep learning method for embedding local structural sites into a numerical vector space. COLLAPSE embeddings were pre-trained at scale using comparisons between evolutionarily related sites across the PDB, enabling them to capture conserved structural and functional features, and we have previously demonstrated that similarity in the embedding space can be used to precisely distinguish between functional sites (42).

Intuitively, if the query protein in question performs a certain enzymatic function, the catalytic residues corresponding to that function should appear near the top of the ranked list of database sites. Detecting enriched functions in this list is analogous to the problem of Gene Set Enrichment Analysis (GSEA) (47), a widely-used method for identifying enriched biological processes in gene expression datasets. Like GSEA, we compute an enrichment score for each function by computing a modified Komolgorov-Smirnov (K-S) statistic. We then define a function-specific empirical p-value to identify statistically significant predictions. The contributing residues for each prediction are identified by mapping the leading-edge subset of database residues back to their nearest correspondences in the query. This algorithm is computationally efficient and can annotate a standard 200-residue protein in 15–20 seconds with no specialized hardware (1 CPU with 4 cores) and less than 10 seconds on a single GPU (compared to 6–8 s for DeepFRI and 12–15 s for CLEAN).

### Accurate function prediction for known enzymes.

**Figure 2.**
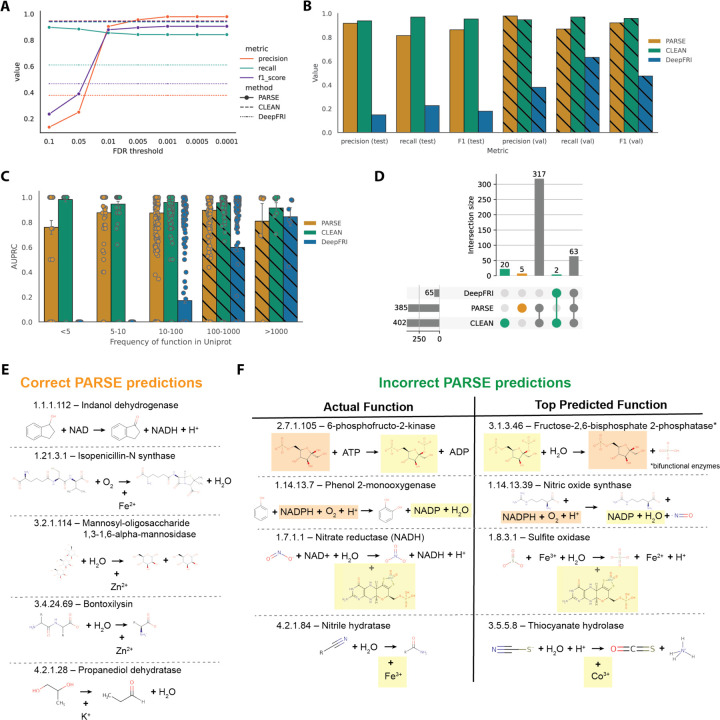
Global prediction performance on enzymes with known function. **(A)** Tuning of FDR-corrected p-value threshold on validation set. Performance in terms of precision, recall, and F1 score at each threshold are compared to PARSE and DeepFRI. **(B)** Precision, recall, and F1 score for each method on held-out test set of rare enzyme classes. Validation set performance is shown in the hatched bars for comparison. **(C)** Function-centric AUPRC for each method depending on the frequency of each enzyme class (EC number) in Swissprot. Each dot represents the AUPRC for a single class, and error bars represent standard error. Enzyme classes with more than 100 examples are in the validation set, shown again using hatched bars. **(D)** Analysis of which enzyme classes are able to be predicted by each method. On the left, the upset plot shows all intersections between the unique EC numbers predicted correctly (AUPRC > 0) for each method. The marginal size of each set is shown by the histograms on each axis. **(E)** EC numbers predicted correctly by PARSE only, including the products, reactants, and cofactors involved in the reaction (orange in (E)). **(F)** Error analysis for four sampled functions which were predicted correctly by CLEAN but not PARSE (green in (E)). Products, reactants, and cofactors which are shared between ground truth and prediction are highlighted in yellow and orange.

We evaluated the performance of PARSE using a dataset of 17,262 known enzymes derived from a non-redundant subset of SwissProt, with corresponding structures predicted by AlphaFold2 (32, 33). We compared our results to CLEAN (state-of-the-art for global function prediction) and DeepFRI (structure-based model with residue saliency mapping). For both baselines, we run the models in inference mode using the provided weights out-of-the box. Importantly, most or all of the proteins in our evaluation dataset have already been seen by these methods in training, inflating their true out-of-distribution performance relative to PARSE. To mitigate this, we selected 3623 proteins with EC numbers represented less than 100 times in SwissProt as a held-out test set for evaluation, expecting that these represent the most challenging cases for supervised models. The remaining 13,639 proteins were used as a validation set to compute empirical score distributions and identify optimal significance thresholds for identifying enzymatic function (Fig. 2A). We find that validation performance plateaus at an FDR-corrected p-value of 0.001, which we choose as a threshold for all future experiments. At this threshold, PARSE performs similarly to CLEAN, with slightly better precision but lower recall, and significantly outperforms DeepFRI in both metrics.

On the test set, we see similar results, with very little performance drop for both PARSE and CLEAN relative to the validation set (Fig. 2B). To gain more insight into the performance on rare enzymes, we measured the function-centric area under the precision recall curve (AUPRC) for each method over all functional classes in the dataset. Even for very rare enzymes with less than five examples in SwissProt, we achieve good performance (mean AUPRC = 0.759), while DeepFRI performance drops to zero (Fig. 2C). CLEAN achieves consistently high performance due to its contrastive learning objective, even with the caveat that even rare enzymes were seen during training.

Next, we examined the errors made by each method to better understand their performance characteristics. For proteins that were not annotated correctly by the top-ranked prediction, we quantified whether the method was predicting a similar function or lower level of specificity (i.e. shared 3^rd^ level or higher of the EC hierarchy), whether it was predicting an entirely different function, or whether it made no predictions at all (Fig. S2). We find that the majority of incorrect predictions made by all three methods do indeed share at least one EC number, and DeepFRI is particularly notable for its lack of specificity, with the majority of predictions correct at the 3^rd^ level but not at the 4^th^ (in many of these cases, the full EC number is present but lower-ranked in the list). Notably, PARSE declines to make a prediction in more cases than both DeepFRI and CLEAN, but when it does make a prediction it is more likely to be correct to the 4^th^ EC level.

We also analyzed which individual functions could be predicted by each method (Fig. 2D). PARSE and CLEAN show high agreement (77.9% of EC numbers) and all three methods agree on a further 15.5%. There were five functions which only PARSE could identify; notably these seem to be enriched for the presence of metal ions as cofactors, which appear in four of these functions (Fig. 2E). There were also 22 functions which PARSE could not annotate correctly (Fig. 2F). Among these misannotations are a group of bifunctional enzymes where only one function is recognized (fructose-6-phosphate-2-kinase–EC 2.7.1.105/fructose-2,6-biphosphatase–EC 3.1.3.46) (Fig. 2F). The remainder of predictions shared either reactants (e.g. ATP, NADPH), products (e.g. ADP, NADP), or cofactors (e.g. metal ions, molybdopterins) with the true catalyzed reaction. This demonstrates the ability of PARSE to detect relevant functional sites even when the precise reaction involved may be more difficult to predict.

### Precise identification of catalytic residues.

**Figure 3.**
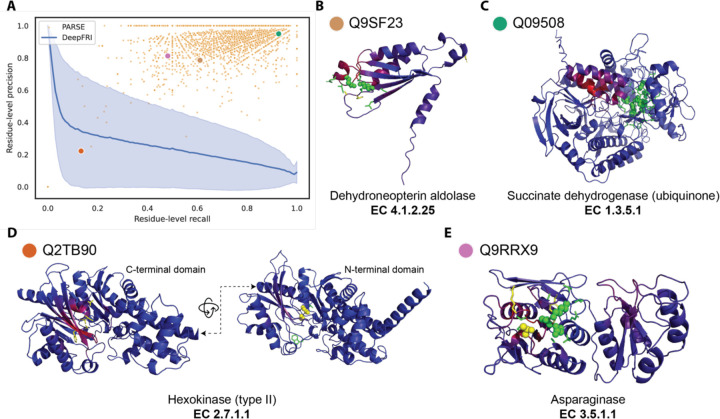
Annotation of enzyme active sites at amino-acid resolution. **(A)** Precision and recall of active site residue identification for all correct predictions in the validation set. Each orange dot represents a single protein, and the four sampled proteins in **(B–E)** are labeled with colored dots. For comparison, DeepFRI performance is represented as a precision-recall curve, where the blue line is the average over all proteins and the shaded error bar is the standard deviation. Four sampled structures, representing active site annotations by PARSE across proteins with diverse performance characteristics and enzymatic activities: **(B)** dehydroneopterin aldolase, **(C)** succinate dehydrogenase, **(D)** type-II hexokinase, and **(E)** asparaginase. In all examples, correctly identified active site residues are shown as green sticks. Correctly identified catalytic residues are shown as green spheres, and catalytic residues which are not identified by PARSE are shown as yellow spheres. Residues annotated by PARSE but not present in the reference site from CSA are shown as yellow sticks. The backbone cartoon is colored by DeepFRI’s gradient-weighted class activation map score, from blue (low) to red (high).

In addition to accurately predicting a protein’s global function, we tested how well PARSE identifies the individual residues involved in carrying out that function. For enzymes, these residues comprise the active site, which we define using the amino acids assigned a catalytic function by CSA and all immediate neighbors within 3.5 Å in the protein structure. For each correct prediction in the held-out test dataset (whether it was the top-ranked prediction or not), we computed the residue-level precision and recall for active site annotation. We compare only to DeepFRI because CLEAN does not produce residue-level predictions. Since DeepFRI produces a quantitative saliency score for each residue instead of a binary prediction, we compare performance for each protein across all possible score thresholds using precision-recall curves. Across the whole dataset, PARSE was able to identify active site residues much more accurately, with residue-level performance of most proteins exceeding that of DeepFRI regardless of threshold (Fig. 3A). Furthermore, PARSE achieves greater precision at equivalent recall in 584 of the 599 test proteins predicted correctly by both methods (Fig. S1). In general, PARSE predictions are more specific than sensitive, with 58.7% of predictions achieving precision > 0.9 and recall > 0.5 but only 7.2% achieving both precision and recall exceeding 0.9 (for comparison, 4.0% and 0.0% of DeepFRI predictions reach these respective benchmarks at any threshold). However, this is partially due to our definition of active sites including both known catalytic residues and neighboring residues which may not be as functionally important. Indeed, we find that recall for detecting catalytic residues alone is significantly greater than recall over the entire active site, suggesting that the majority of residues missed by PARSE are non-catalytic (Fig. S3).

To highlight the benefits of PARSE’s residue-level explainability for protein function prediction, we show four examples sampled from a range of performance characteristics and EC classes (Fig. 3B–E). Figure 3B shows an example where we achieve only moderate precision and recall over the entire active site, but both the catalytic Lys and Glu residues are correctly identified. Some proteins, such as the succinate dehydrogenase shown in Figure 3C, are annotated even more accurately—all eight catalytic residues are detected along with their closest neighbors. In both examples, the saliency predicted by DeepFRI is noticeably more diffuse and not centered around the catalytically active residues. Notably, in the latter case DeepFRI focuses instead on the binding site of the FADH cofactor, which is important mechanistically but not specific to this enzyme, being shared by all FAD-dependent flavoproteins.

In some cases, predictions which seem like misannotations may actually provide additional insight into the enzyme’s function and the limitations of existing databases. For example, Figure 3D shows a hexokinase enzyme with two functional domains. Only the catalytic residues in the N-terminal domain were identified by CSA’s homology search, while both DeepFRI and PARSE correctly identify the equivalent residues in the C-terminal domain (representing CSA false negatives), resulting in reduced precision and recall. In another case, shown in Figure 3E, PARSE misses the catalytic Thr16 residue in a putative asparaginase enzyme. However, this protein is also notably missing a key tyrosine (Tyr25 in reference PDB 3eca) that should interacts with Thr16, suggesting that this protein may not in fact be catalytically active. The EC number was assigned to this protein in Swissprot based on homology, demonstrating the benefit of using local representations which capture the complex atomic microenvironment around each residue.

### Scaling annotation to the full human proteome.

The AlphaFold Structure Database contains high-quality predicted structures for the proteomes of 48 organisms (33), offering an opportunity for structure-based functional annotation. We applied PARSE to 21,575 proteins in the human proteome to evaluate its ability to make functional annotations at scale. The FDR cutoff of 0.001 tuned on the validation set resulted in 17,761 functional predictions for 8195 unique proteins. We observed that on this dataset, certain functions were predicted far more often than expected based on their known prevalence, even with the function-specific significance correction. Among these, almost half of the residues identified as functional had no overlap with the reference catalytic residues (Fig. S4A). We find that these spurious predictions are driven largely by low-complexity structures in the AlphaFoldDB (see Fig. S4B for examples), which are highly non-specific and match many different reference structures. Because Swissprot is enriched for higher-complexity proteins, this type of spurious hit is not captured by our background distribution. Therefore, to increase the specificity of our proteome-wide predictions, we implement two filters: (1) at least 75% of reference catalytic residues must be identified by PARSE in the query structure, and (2) the all-atom RMSD between aligned catalytic residues of the two structures is less than five angstroms. The latter condition also requires that at least two catalytic residues must match the reference. These conditions reduced the number of predictions to 1396, representing 1311 unique proteins.

**Figure 4.**
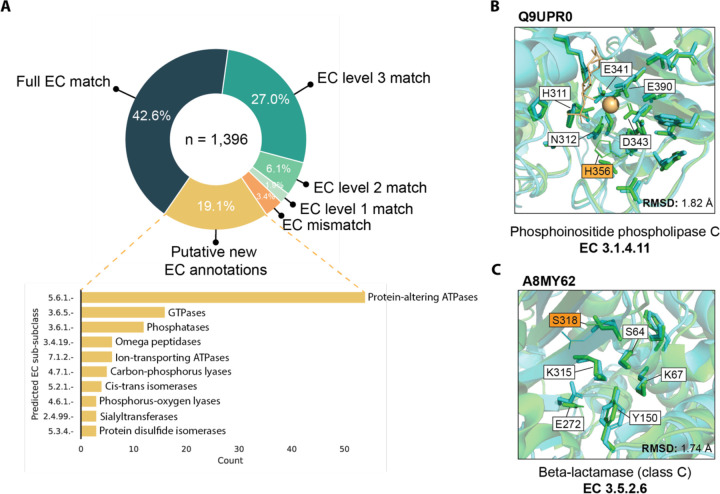
Expanding annotation coverage in the human proteome. **(A)** Comparison of PARSE predictions for AlphaFold structures in the human proteome to EC number annotations in Uniprot, where available. Proteins are labeled as *EC mismatch* if the prediction does not match known annotations at any EC level, and *putative new EC annotations* are proteins with no EC numbers assigned in Uniprot. For these new hypotheses, we show the top 10 predicted enzyme classes at the third EC level. **(B)** Likely inactive PI-PLC with mutant catalytic residue H356T correctly not annotated by PARSE, and **(C)** putative class-C beta-lactamase predicted by PARSE. For both examples, reference structures from PDB are shown in green and query structures predicted by AlphaFold are shown in cyan. Residues identified as functional by PARSE are shown as sticks, and residues aligned to CSA residues but not annotated by PARSE are shown as lines. Catalytic residues are labeled using PDB numbering, and mismatches between query and reference are highlighted in orange. Proteins are aligned and RMSD is computed using catalytic residues only, including both backbone and side chain atoms.

Among these predictions, 69.6% matched an EC number assigned in Uniprot to at least the third EC level (i.e. X.X.X.-), while a further 8.0% matched at either the first or second level (Fig 4A). Only 47 predictions did not match any EC numbers; 12 of these resulted from an EC number transfer that produced a mismatch between Uniprot and CSA annotations, while the remainder are either close homologs or bind similar ligands in the active site. The remaining 266 predictions correspond to proteins with no EC number in Uniprot, representing putative new annotations. The majority of these are ATPases (notably myosins, kinesins, and chaperonins), G-protein GTPases, and phosphatases (notably HSP70 heat-shock proteins), reflecting the ubiquity of these protein families in cellular processing. Most of these are also well-annotated in Uniprot but simply missing an EC number annotation, serving as positive controls and validating the ability of PARSE to identify missing annotations in sequence databases.

We highlight two examples from the human proteome to showcase the utility of PARSE’s residue-level explainability and high functional specificity relative to existing methods. The first example, Q9UPR0, was annotated as a phosphoinositide phospholipase C (PI-PLC) due to high active site homology (Fig. 4B). However, it is likely inactive due to the substitution of a threonine residue for the catalytic histidine (His486 in Q9UPR0), an important feature which could not be detected by global methods. The second example, A8MY62, is much more sparsely annotated, with an assigned label of “putative beta-lactamase-like 1” (Fig. 4C). Beta-lactamases are a diverse class of enzymes with several subclasses (A, B, C, and D) which are further subdivided by catalytic mechanism and substrate specificity. All beta-lactamases share a single EC number (3.5.2.6), so they cannot be distinguished by existing methods for enzyme prediction which rely on EC number alone to define labels. PARSE, on the other hand, can predict any unique catalytic mechanism in CSA, allowing it to assign enzyme function with greater specificity. In this case, PARSE identifies A8MY62 as a class C beta-lactamase with no significant hits to other classes. Although a serine residue (Ser318 in the reference structure) is missing from the active site, mutagenesis studies have shown that mutations at this position do not affect the specificity of the enzyme (48, 49). The explainability of PARSE’s predictions thus facilitate confident assessments and provide biological intuition for computational functional predictions.

### Functional hypotheses for novel folds in the dark proteome.

Protein research is strongly biased towards common and well-studied proteins, while the biological functions of thousands of others remain poorly understood (29). A recent clustering analysis of the entire AlphaFoldDB using Foldseek (50) identified over 40,000 clusters which could not be annotated using similarity to structures from known domain families. We hypothesized that PARSE’s ability to discover conserved local functional sites even with low fold-level similarity would make it ideally suited to discover novel enzymes in this dataset. Using the same filtering procedure as described for the human proteome to identify high-confidence hits, we annotated 34,015 representative structures from the dark proteome. This process predicted 44 putative novel enzymes from 17 different third-level EC classes (Fig S5A). A full list of these predictions is provided in Supplementary Data 1, and two examples are visualized in Fig S5B–C.

**Figure 5.**
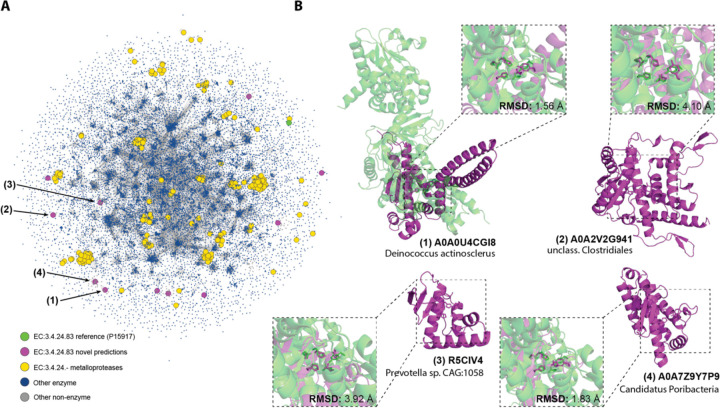
Annotation of dark proteome reveals novel metalloprotease folds. **(A)** Structural similarity of putative novel metalloproteases relative to the universe of known enzymes. New predictions are shown in purple, the CSA reference for EC number 3.4.24.83 in green, and other known metalloproteases in yellow. Blue dots are known enzymes in Swissprot, and edges are shown between proteins with similarity of less than 0.001 by Foldseek e-value. **(B)** Examples of four novel predictions (shown in purple), each aligned with the CSA reference structure (PDB 1PWV; green) using all atoms in the five catalytic residues (His686, Glu687, His690, Tyr728, and Glu735).

Interestingly, a large number of predictions belong to metalloprotease families (EC 3.4.24.-). In particular, 11 were predicted to belong to the EC number 2.4.24.83, a zinc-dependent endopeptidase which cleaves the N-terminus of mitogen-activated protein kinase kinases (MAPKKs). This enzyme and its homologs are key components of many bacterial toxins, making its inhibition attractive for therapeutic purposes (51). The predictions made by PARSE come from diverse bacterial species and exhibit unique structural folds, none of which show significant similarity to any known metalloprotease (Fig. 5A). In Figure 5B, we show global and local active site structure for four of these predictions superimposed on the reference PDB structure based on the conformation of the five key catalytic residues. All predictions show high active site conservation despite the divergence in global fold, strongly suggesting a shared catalytic mechanism.

## Discussion

To improve widespread acceptance and trust in artificial intelligence in biology, it is important for methods to provide not only accurate predictions, but also explanations that correspond to biological intuition. In this work, we propose a new approach to protein function annotation that combines the advantages of pre-trained protein representations with prior biological knowledge and statistical methods to improve explainability while retaining high predictive performance. In contrast to standard supervised learning approaches which start with global classification and then attempt to explain these predictions *post-hoc*, PARSE is a bottom-up approach that starts by identifying putative functional sites at the residue level before aggregating predictions over the entire protein. This formulation is explainable by construction, since any global prediction can be traced back to each contributing residue, and improves over methods which rely on single site-level comparisons because it combines signal over multiple sites which may have individually moderate similarity.

A major strength of the PARSE algorithm is its modularity and flexibility; each component can be easily adapted based on the biological task. For example, a new reference database could be constructed for or any problem where residue-level knowledge bases exist (e.g. ligand-binding sites, post-translational modifications), or new functions could be added manually based on new experimental data. Adjusting the significance threshold controls precision and recall depending on which is more important based on the task at hand. The local representation could also be adapted for other data types; while COLLAPSE is the primary embedding method for local protein sites, any pre-trained representation could be used for other data formats. Finally, the GSEA-like scoring function could be replaced with any statistical method which returns enrichment scores for each class and the key residues which contribute to the prediction. Improvements here may help to increase statistical power and reduce the influence of low-complexity structures which cause false positives in our proteome-wide scans. This is a known weakness of the K-S test when used in a pre-ranked setting, which tends to overestimate significance for sets with high internal correlation between elements (52). Our function-specific empirical significance calculation largely addresses this problem, but may still produce false positives for proteins that are outside the background distribution (e.g. AlphaFold predictions for the dark proteome).

The most significant limitation of PARSE is its reliance on a high-quality database of residue-level labeled data. The Catalytic Site Atlas is an excellent resource for this purpose, but it is limited to 940 enzymes and has not been updated for several years. This highlights the importance of expanding site-level as well as global protein annotation databases as biological knowledge increases. Importantly, since PARSE requires only one reference example to make predictions, it is relatively straightforward to curate larger datasets without requiring high-throughput experiments. As methods for extracting and synthesizing knowledge from across biomedical literature improve, we anticipate that large-scale databases will become more widespread, expanding the coverage of site-based methods such as PARSE for new function discovery.

For enzyme function prediction, PARSE recapitulates known Swissprot annotations much more accurately than DeepFRI, the best-performing existing method which provides residue-level explanations. The improvement is especially notable for rare and understudied enzyme classes, an important characteristic which can be attributed to the one-shot nature of PARSE’s database comparisons. The best-in-class global method, CLEAN, also has few-shot ability due to its contrastive learning objective. Although it performs better than PARSE on our rare enzyme dataset, it is important to note that the publicly available implementation of CLEAN was pre-trained on a 100% non-redundant clustering of Swissprot, so even the rare enzymes are in-distribution for this model.

At the amino acid level, we perform the largest-scale quantitative evaluation to date of residue-level performance for machine learning based protein function prediction models. We find that residue-level annotations provided by PARSE correspond much more accurately to the catalytic site of the enzymes than DeepFRI’s class activation mapping approach. This is in agreement with previous studies which note the pitfalls of *post-hoc* gradient-based explainability methods (45, 46). Gradient-based methods are also become increasingly unsuitable as large-scale foundation models (53) become increasingly widespread in biology, since such models are run almost exclusively in inference mode and often do not provide access to internal model weights. We anticipate that methods such as PARSE, which combine pre-trained embeddings with prior biological knowledge and interpretable statistics, will be critical for making explainable and trustworthy predictions in this new paradigm.

The release of AlphaFoldDB provides an unprecedented opportunity to apply structure-based predictors to discover new biological functions at proteome scale. On this largely unexplored dataset, especially the entirely novel folds in the dark proteome, the residue-level explainability of PARSE is especially important for evaluating predictions, as we show through several illustrative examples. Most notably, we discover strong evidence for several new bacterial metalloproteases which have highly divergent structures and sequences but retain a strongly conserved active site. These findings illustrate the potential of local representations combined with large structural databases to discover new functional insights, which may help our understanding of pathogenic processes and aid in the development of more potent and specific therapeutics. As protein structure predictors improve and databases continue expand to hundreds of millions of metagenomic proteins (22), we expect that methods such as PARSE will become even more powerful tools for biological discovery.

## Materials and Methods

### Reference database construction

Our reference database consists of the manually curated residue-level annotations for enzymes in the Catalytic Site Atlas. We extract the relevant chain and catalytic residue identifiers from the reference PDB entry for each enzyme class. Since the average number of catalytic residues for each structure is less than five, which is not enough on its own to achieve good statistical power in large-scale searches, we expand the enzyme active site to include all residues which have at least one atom within 3.5 Å of a catalytic residue. This threshold was chosen to capture any residue that may interact with a catalytic residue (e.g. via hydrogen bonding). We remove all ligands, waters, and other heteroatoms from the reference chain. Then, we embed the structural microenvironment surrounding each active site residue to a 512-dimensional numeric vector using COLLAPSE, which considers all atoms within a 10Å radius of the pre-defined functional center of each amino acid (42). The result of this process is a database consisting of 26,157 residues corresponding to 939 unique functional sites.

### Evaluation dataset creation and processing

We evaluated on AlphaFold predicted structures for known enzymes in SwissProt, starting with the sequence homologs provided by CSA, which are identified by searching each reference sequence against Uniprot using PHMMER (54) with an e-value cutoff of 1 × 10^−6^. Conserved catalytic residues in these alignments are then annotated to serve as a ground truth for residue-level predictions. Since these results are based on sequence similarity, there are many false positives (e.g. proteins from related families with different catalytic mechanisms). Therefore, to create a “gold-standard” dataset for evaluation we included only proteins with a curated SwissProt EC number that perfectly matches the EC number for the reference CSA entry. We then redundancy-reduced this dataset using 50% sequence identity clusters from Uniref50 (55) to ensure that each protein belongs to a different sequence cluster. We also removed all proteins which share a sequence cluster with any protein in the reference database. This process resulted in 17,262 unique proteins representing 17,779 total function annotations. To create a held-out test set which would not be used for tuning the significance threshold, we binned proteins by the frequency of their corresponding enzyme classes in SwissProt. All enzymes with at least 100 examples were used for validation (269 unique functions) and the remainder were reserved for testing (425 unique functions). For all datasets derived from AlphaFoldDB (Swissprot validation and test, human proteome, and dark proteome), the environment around every residue with high or very high confidence (*pLDDT* ≥ 70) was embedded using COLLAPSE and stored along with corresponding metadata (e.g. Uniprot ID, residue IDs, pLDDT). Links to download these pre-computed datasets are provided along with the code in our Github repository.

### PARSE implementation details

The PARSE algorithm consists of three main steps, outlined here in detail and shown in Figure 1.

1. **Embed input protein.** Every residue of the input structure is embedded using COLLAPSE (42), using the same parameters as in the construction of the reference database. If the input structure is an AlphaFold predicted structure, we only consider residues with *pLDDT* ≥ 70 to reduce the influence of low-confidence structural regions.
2. **Rank reference residues by similarity to input protein.** First, we compute the pairwise cosine similarity between the database embeddings and the input protein embeddings. Then, for each database site we identify the maximum similarity to any residue in the query. Database sites are then sorted by this maximum similarity to produce the final ranked list. This process also produces the mapping between database sites and the nearest residue in the query which is used to compute final residue-level annotations.
3. **Identify enriched classes and key functional residues.** We compute an enrichment score (ES) statistic for each function class *F* by walking down the ranked list and increasing or decreasing a running sum statistic *S* depending on whether the database residue is in *F* or not in *F*, respectively. We use the same increment and decrement formulas as in GSEA (47) to compute *S*, and the ES is similarly calculated as a weighted Komolgorov-Smirnov statistic using the maximum deviation of *S* from zero. The raw ES should not be used to directly rank functional classes due to the differences in the null distribution of scores within each class, necessitating the calculation of class-specific significance scores (52). In standard pre-ranked GSEA, statistical significance is assessed by permuting the gene labels; however, this is known to overreport significance when there is high correlation within gene sets. We observe the same phenomenon for our dataset, so we instead estimate significance using a function-specific empirical ES distribution. Specifically, for each function class we measure the ES over all proteins in our validation and test datasets that are not annotated with that function in Swissprot. The empirical p-value for a new enrichment score *s* is then computed as $p=\frac{\sum_{i}^{\left|D\right|}\left(s>d_{i}\right)}{\left|D\right|}$, where $d_{i}\in D$ are the individual ES over the background distribution *D*. This approach is similar in spirit to the permutation of association scores proposed for multi-sample GSEA by Tian et al. (56) and significantly improves the sensitivity and specificity of the resulting predictions. To correct for multiple hypothesis tests, we control false discovery rate (FDR) using the Benjamini-Hochberg procedure.

### Baseline methods

Our goal was to compare PARSE to existing methods out-of-the-box, as they would be used by practitioners. Therefore, we used the inference scripts and pre-trained model weights provided in the Github repositories for DeepFRI (https://github.com/flatironinstitute/DeepFRI) and CLEAN (https://github.com/tttianhao/CLEAN) directly. For DeepFRI, we pre-processed all PDB files to produce distance maps and sequence embeddings and predicted EC numbers using the default model architecture: three MultiGraphConv convolutional layers with dimension 512, followed by a linear encoder of dimension 1024. Final predictions are assessed using the default predicted probability cutoff of 0.1. For CLEAN, we pre-process the dataset into fasta files by unique chain, use the default *split100* pre-trained model weights, and make predictions using the maximum separation procedure. Note that the set of possible EC number labels DeepFRI is trained on are not identical to those used for CLEAN; this is reflected in the baseline comparison results, particularly where less specific labels are preferred by DeepFRI (i.e. third level EC number). Since PARSE uses enzyme class definitions defined by catalytic mechanism in CSA, which is more specific than EC numbers in some cases. For all baseline comparisons, we convert the CSA class predicted by PARSE to its corresponding EC number.

### Human and dark proteome datasets

The human proteome dataset was downloaded from AlphaFoldDB (https://www.alphafold.ebi.ac.uk/download) on July 20, 2021. The Uniprot accessions the dark proteome were derived from the data provided by Barrio-Hernandez et al (2023) (35). We used the reference structures for each dark cluster with average pLDDT > 90 downloaded from the AlphaFoldDB website on October 21, 2022. All predicted structures for both human (n = 21,575) and dark (n = 34,015) proteomes were processed as described for the Swissprot evaluation dataset, removing structures that had no high-confidence residues and embedding using COLLAPSE.

### Active site alignment to reference structures

We use structural conservation of the active site as one piece of evidence to support a functional prediction and filter down proteome-scale results. We quantitatively evaluate conservation using root-mean-square deviation (RMSD) between all matching catalytic residues in the active site. To compute this, we identify PARSE annotations with an exact amino acid match to the corresponding catalytic residue in CSA and extract the 3D coordinates of all atoms (including side chain and backbone) in these residues from both reference and query structures. We then align these sets of coordinates using the Kabsch algorithm (57) and compute RMSD between all atoms.

## Supplementary Material

Supplement 1
