# Supplementary material for: Protein functional site annotation using local structure embeddings
Source: bioRxiv. 2025 Jun 25:2023.10.13.562298. Originally published 2023 Oct 16. Preprint. [Version 2] doi: 10.1101/2023.10.13.562298 (PMC10614799; doi:10.1101/2023.10.13.562298)
Supplement: Supplement 1 [file media-1.pdf]

Protein functional site annotation using local structure embeddings

Alexander Derry, Alp Tartici, Russ B. Altman

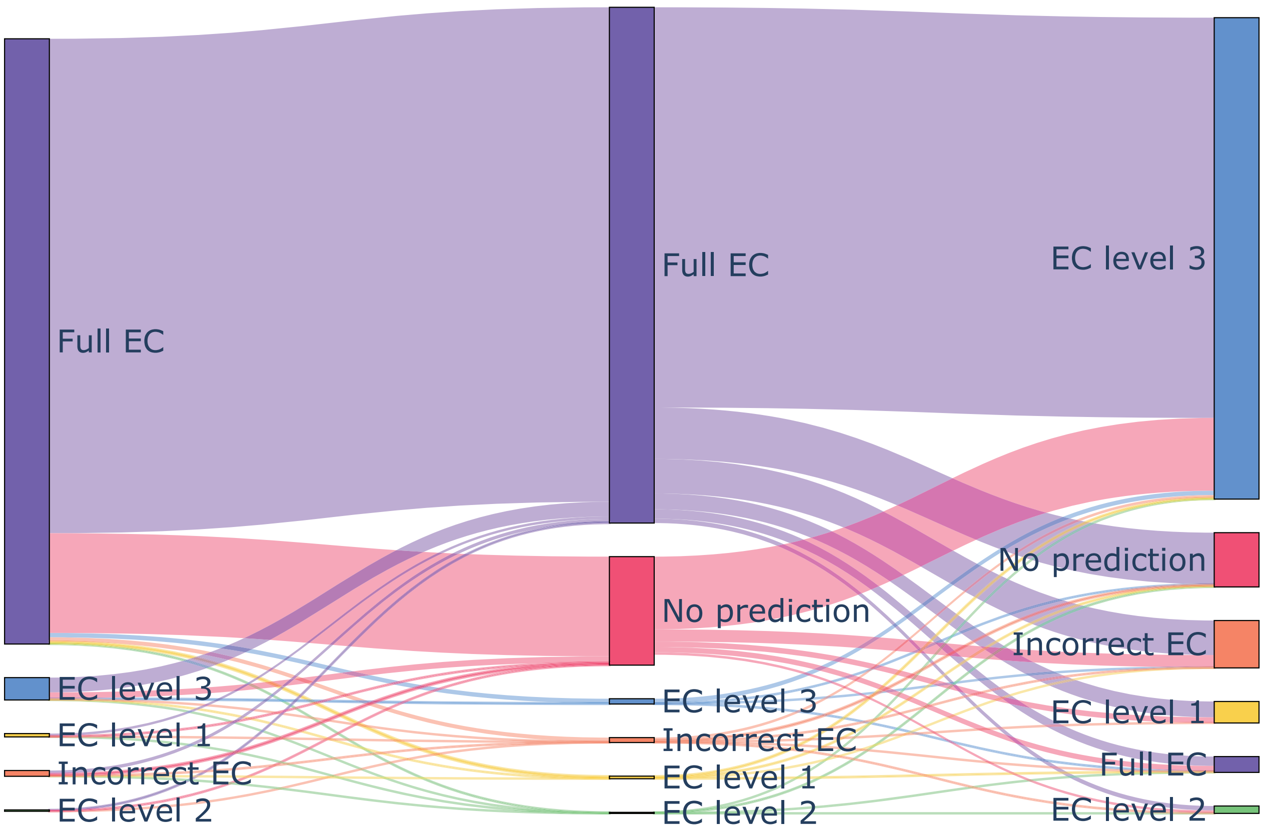

Fig. 1. Comparison of top-ranked predictions for each method. Errors are classified into buckets depending on the most specific level of the EC hierarchy they predict correctly (if any).

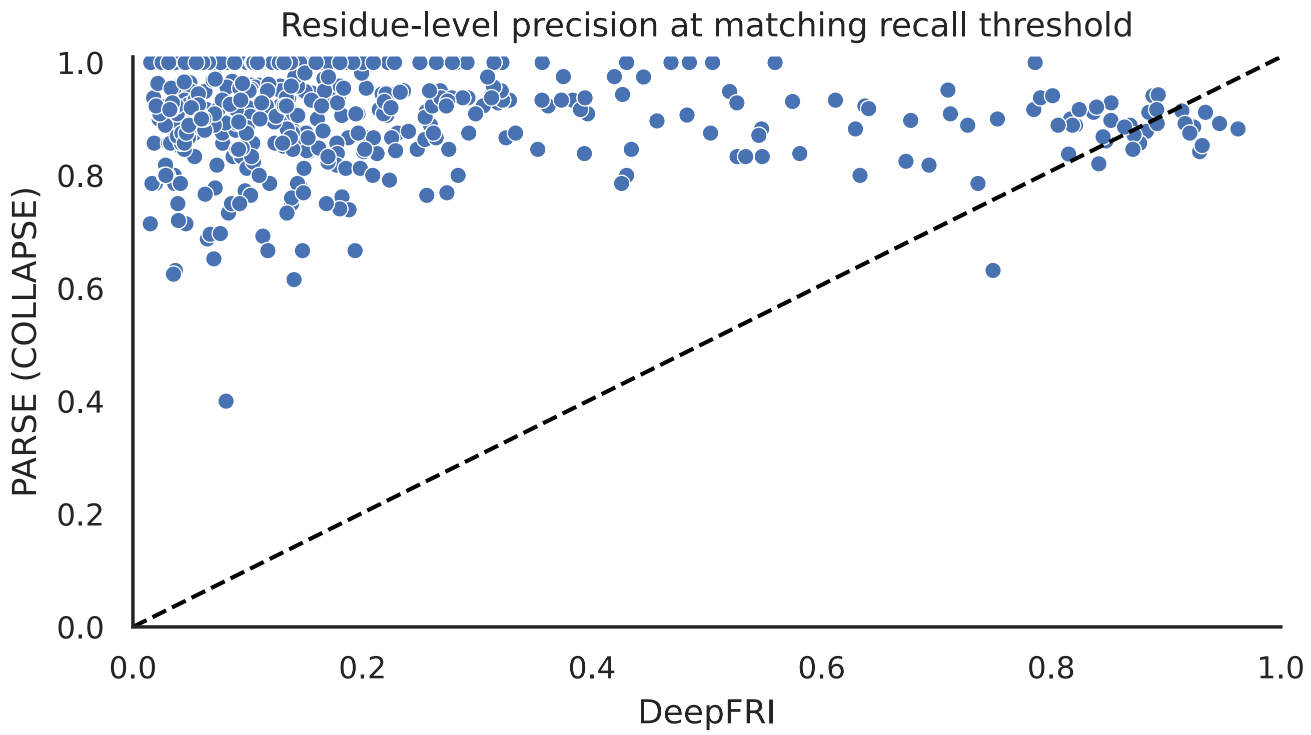

Fig. 2. Comparison of residue-level precision for active site prediction. Each dot represents a single protein which was predicted correctly by both PARSE and DeepFRI (n = 599). Precision for DeepFRI was calculated using the threshold corresponding to the recall nearest to the recall achieved by PARSE for that protein.

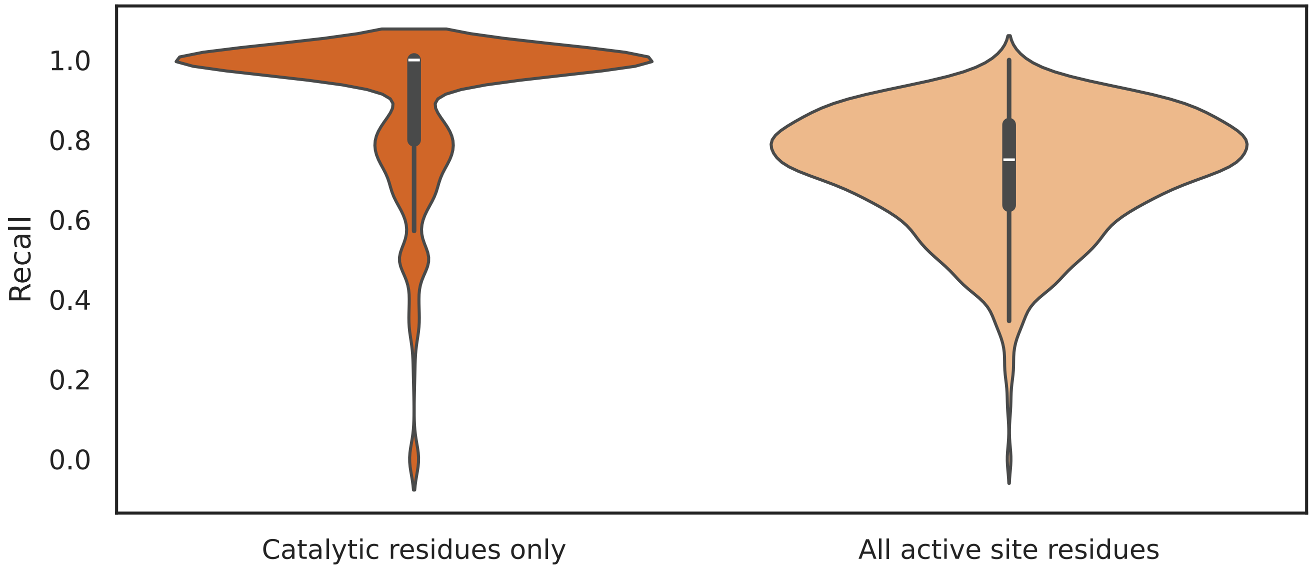

Fig. 3. Residue-level recall of PARSE on held-out test set for catalytic residues defined in CSA compared to all residues in the active site (catalytic residues plus neighboring residues within 3.5 Å)

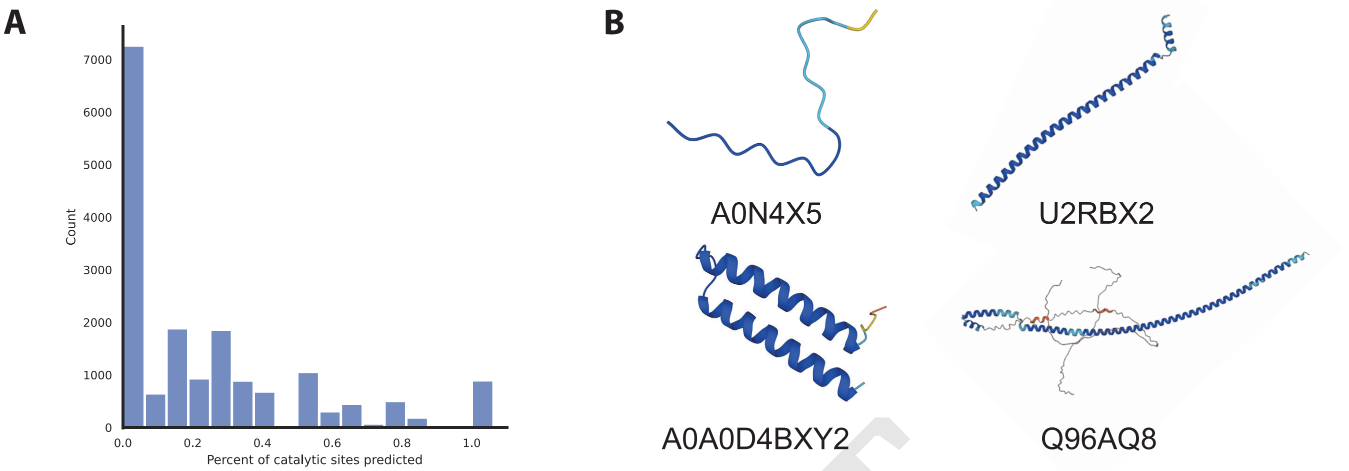

Fig. 4. (A) Percent of reference catalytic sites identified for each functional prediction in human proteome. (B) Examples of low-complexity predicted structures in the human proteome which have high average pLDDT. Such structures spuriously match many reference proteins, resulting in false positives.

DRAFT

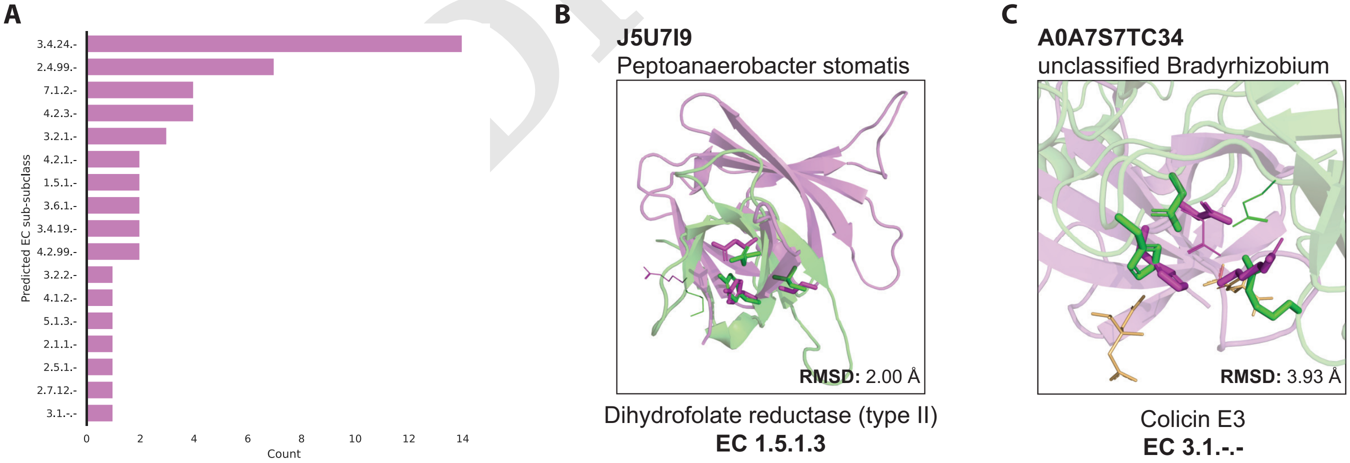

Fig. 5. Non-metalloprotease enzymes predicted in the dark proteome. Reference PDB structure is shown in green and the query structure is shown in purple. Catalytic residues from CSA identified by PARSE are shown as sticks, and those that do not match or are not found by PARSE are shown as lines. (A) EC number distribution for dark proteome predictions with greater than 75% of reference catalytic residues matching and less than 5 Å RMSD between catalytic residues. (B) Example prediction for a DFHR enzyme, with 3 out of 4 catalytic residues matching the CSA reference with low RMSD. The final catalytic residue, Lys32, is replaced by another basic amino acid (Arg) in the same position. (C) Example prediction for colicin E3, a ribonucleolytic enzyme. The key Glu-His-Asp catalytic triad is present, albeit in a slightly different orientation, despite highly divergent global folds, suggesting that this protein is an enzyme with a similar mechanism to colicin E3.

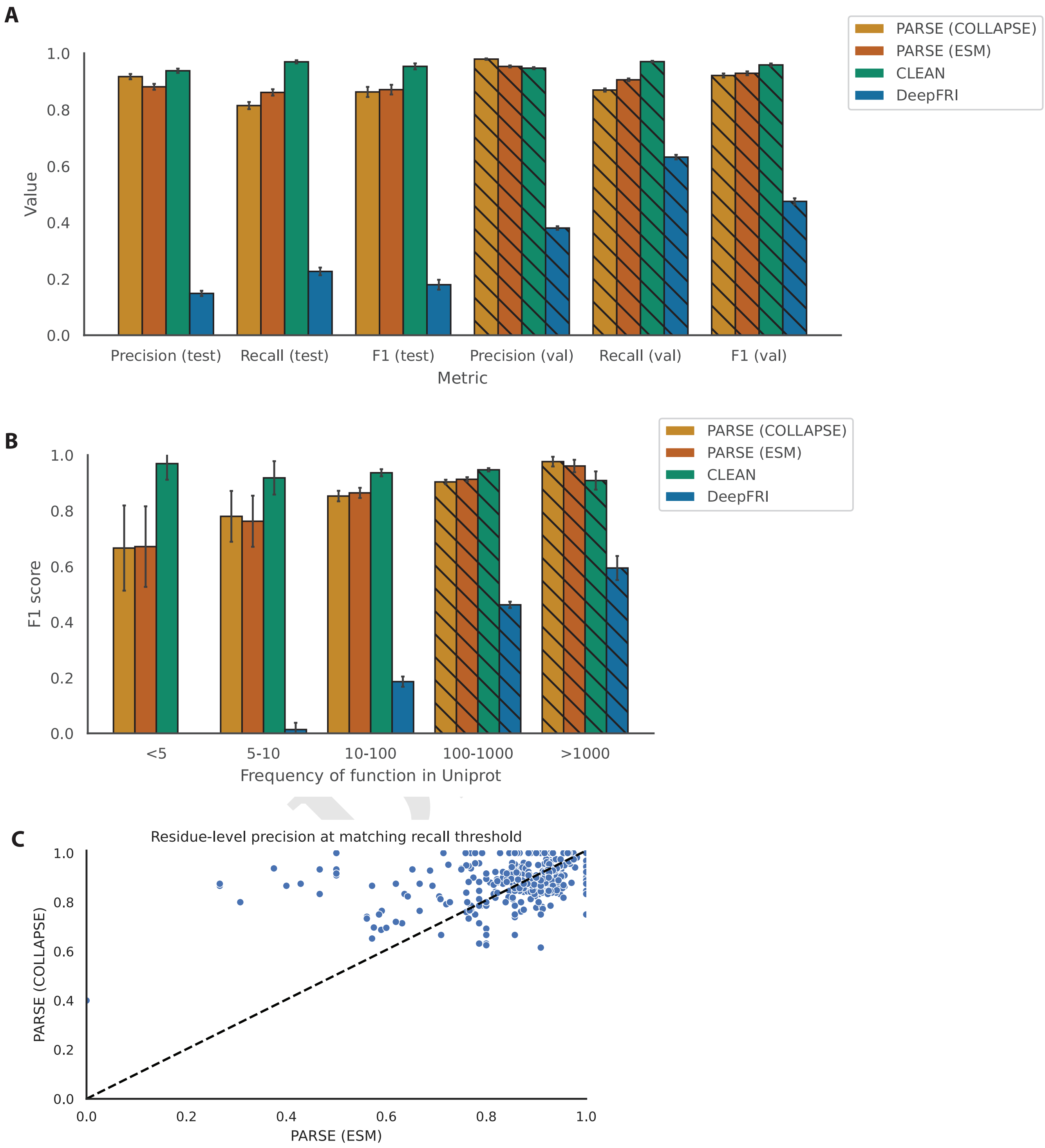

Fig. 6. Comparison between PARSE implemented with COLLAPSE (local structure microenviroment embeddings) and ESM-2 (residue-level embeddings from protein language model). We computed residue-level embeddings from the final layer of the ESM-2 650M parameter model applied to each of protein sequence and used these as a drop-in replacement for COLLAPSE embeddings in the PARSE algorithm. The FDR-corrected p-value cutoff for the PARSE-ESM predictions was 0.0005, tuned on the validation set as described in the main manuscript. (A) Precision, recall, and F1 score for each method on held-out test set of rare enzyme classes, plotted identically to Fig. 2B. (B) F1 score for each method by frequency of each enzyme class (EC number) in UniprotKB (Swissprot), plotted identically to Fig. 2C. (C) Comparison of residue-level precision for active site prediction on the test set between PARSE-COLLAPE and PARSE-ESM. Each dot represents a single protein, analogous to Fig. S1.

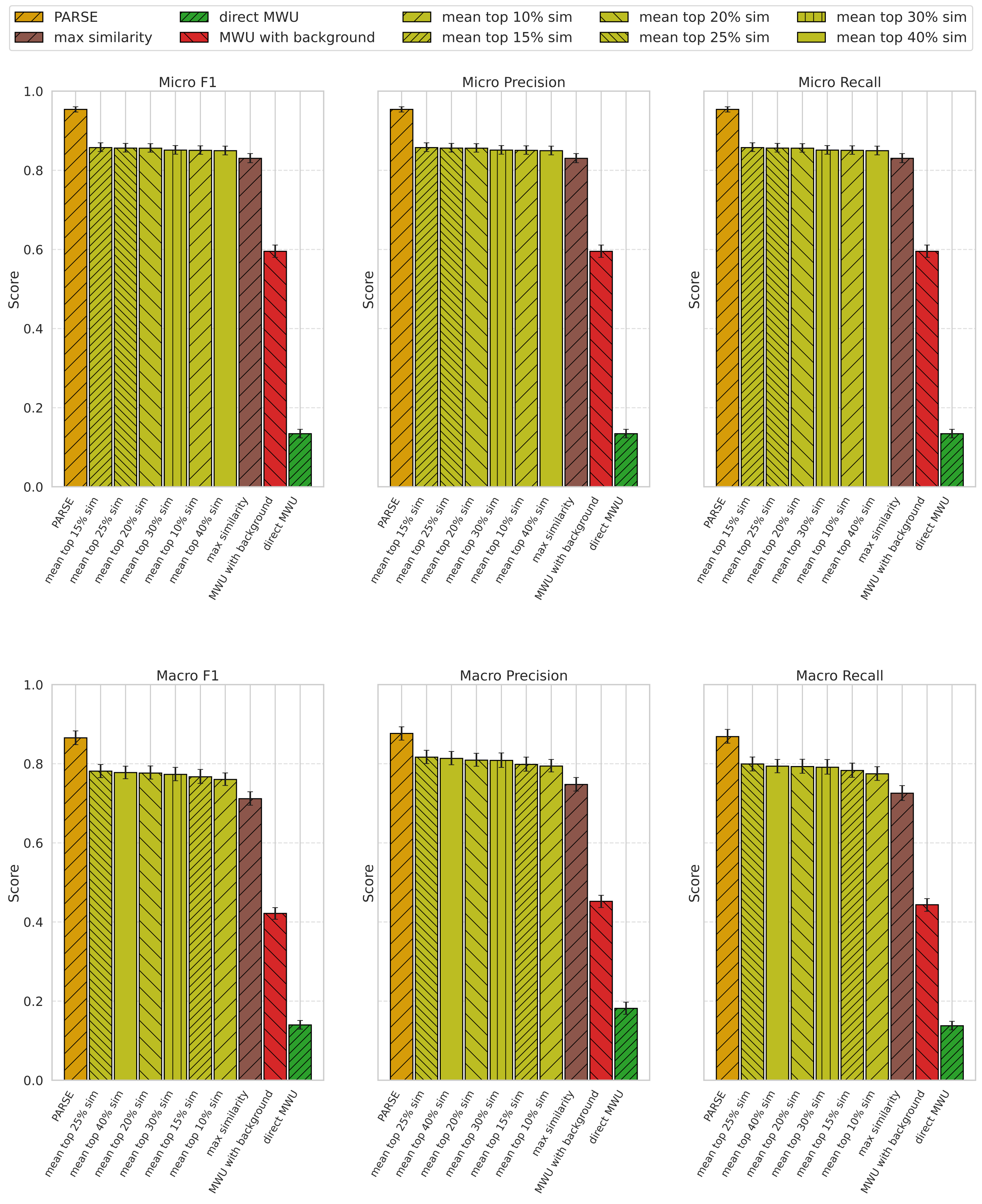

Fig. 7. Performance comparison of PARSE against four different types of COLLAPSE-based prediction baselines using six different metrics. Each baseline ablates a different aspect of the post-COLLAPSE statistical analysis for EC number prediction. FDR cutoff for PARSE was set to 0.001.

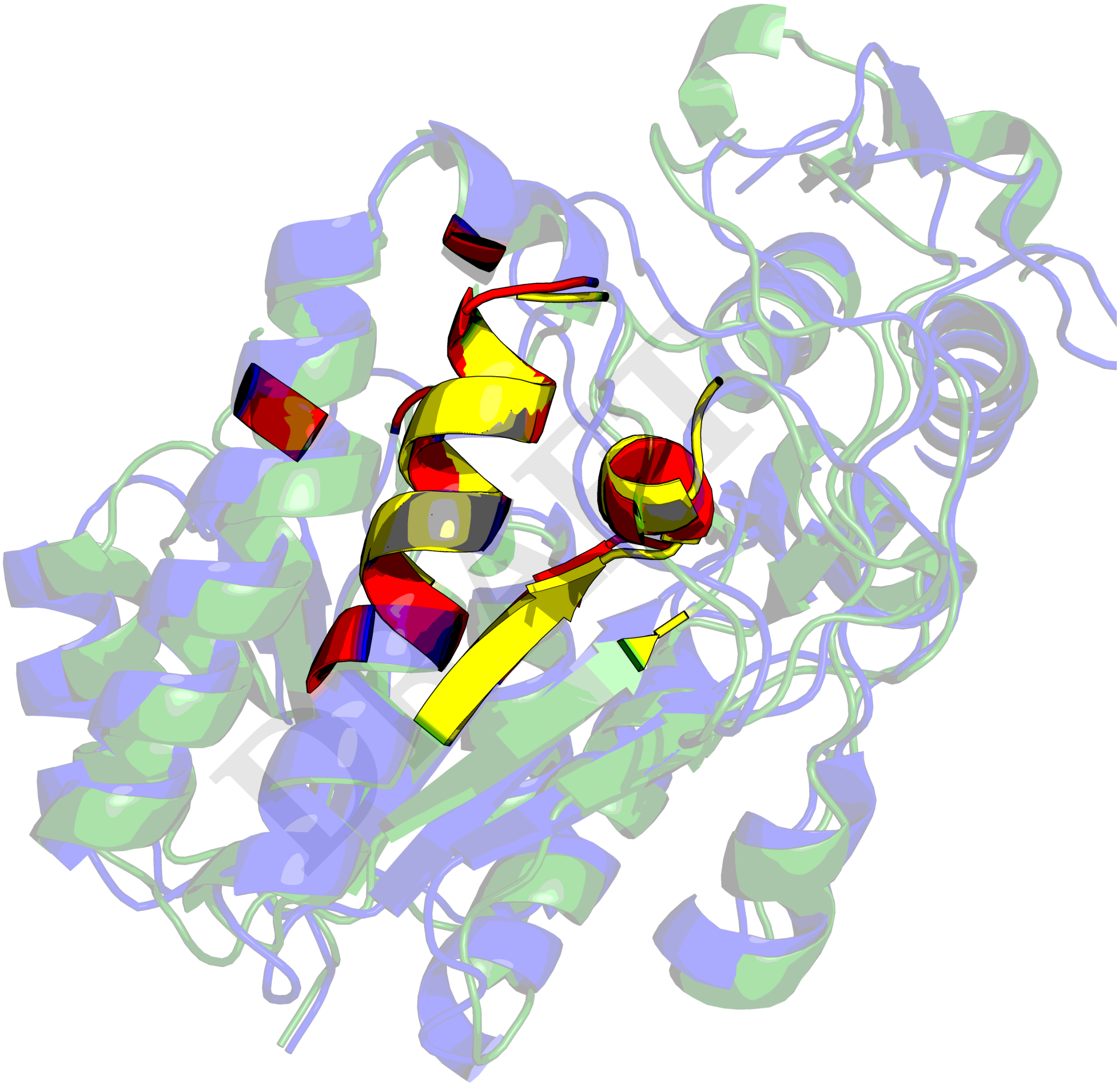

Fig. 8. An example of high global structural alignment between two proteins whose EC numbers diverge at the highest level (5.1.3.2 and 4.2.1.47), both correctly predicted by PARSE. The chain in green is GDP-mannose 4,6-dehydratase (Q51366), with its prediction hit residues highlighted in yellow. The chain in blue is UDP-glucose 4-epimerase (Q14376), with its prediction hits residues in red, partially overlapping with those of the former. The TM-scores for global structural alignment are 0.83 and 0.89, normalized by the respective sequence lengths. The structures are taken from AlphaFold2 predictions.

Table 1. Predictions on proteins with identical SCOP code and high global structural similarity but divergent EC number (86% accuracy, mispredictions colored in red).

UniProt ID	SCOP	Functional label	Prediction	EC Label	Prediction
Q9SUM3	b.43.4.0	NADPH-hemoprotein reductase	NADPH-hemoprotein reductase	1.6.2.4	1.6.2.4
P14779	b.43.4.0	NADPH-hemoprotein reductase	cytochrome P450 (BM-3)	1.6.2.4	1.14.14.1
Q2YN92	b.43.4.0	riboflavin synthase	riboflavin synthase	2.5.1.9	2.5.1.9
P81445	b.6.1.3	nitrite reductase (copper type)	nitrite reductase (copper type)	1.7.2.1	1.7.2.1
Q70KY3	b.6.1.3	laccase	laccase	1.10.3.2	1.10.3.2
P08310	c.1.11.2	muconate cycloisomerase (syn)	muconate cycloisomerase (syn)	5.5.1.1	5.5.1.1
P42206	c.1.11.2	glucarate dehydratase	glucarate dehydratase	4.2.1.40	4.2.1.40
Q8LAH7	c.1.4.1	12-oxophytodienoate reductase	NADPH dehydrogenase	1.3.1.42	1.6.99.1
Q4D3W2	c.1.4.1	dihydroorotate dehydrogenase (fumarate)	dihydroorotate dehydrogenase (fumarate)	1.3.98.1	1.3.98.1
Q9FUP0	c.1.4.1	12-oxophytodienoate reductase	12-oxophytodienoate reductase	1.3.1.42	1.3.1.42
Q65CX5	c.1.8.3	arabinogalactan endo-1,4-beta-galactosidase	arabinogalactan endo-1,4-beta-galactosidase	3.2.1.89	3.2.1.89
O22317	c.1.8.3	glucan endo-1,3-beta-D-glucosidase	licheninase (glycosyl hydrolase 17 family)	3.2.1.39	3.2.1.73
P28997	c.2.1.0	glutamate dehydrogenase	glutamate dehydrogenase	1.4.1.2	1.4.1.2
Q13630	c.2.1.0	GDP-L-fucose synthase	GDP-L-fucose synthase	1.1.1.271	1.1.1.271
E8MF10	c.2.1.0	UDP-glucose 4-epimerase	UDP-glucose 4-epimerase	5.1.3.2	5.1.3.2
Q86A17	c.2.1.0	6,7-dihydropteridine reductase	6,7-dihydropteridine reductase	1.5.1.34	1.5.1.34
Q14376	c.2.1.2	UDP-glucose 4-epimerase	UDP-glucose 4-epimerase	5.1.3.2	5.1.3.2
P95780	c.2.1.2	dTDP-glucose 4,6-dehydratase	dTDP-glucose 4,6-dehydratase	4.2.1.46	4.2.1.46
Q51366	c.2.1.2	GDP-mannose 4,6-dehydratase	GDP-mannose 4,6-dehydratase	4.2.1.47	4.2.1.47
P42556	c.2.1.2	pteridine reductase	pteridine reductase	1.5.1.33	1.5.1.33
P26391	c.2.1.2	dTDP-glucose 4,6-dehydratase	dTDP-glucose 4,6-dehydratase	4.2.1.46	4.2.1.46
O60547	c.2.1.2	GDP-mannose 4,6-dehydratase	GDP-mannose 4,6-dehydratase	4.2.1.47	4.2.1.47
P09417	c.2.1.2	6,7-dihydropteridine reductase	6,7-dihydropteridine reductase	1.5.1.34	1.5.1.34
Q9KQG2	c.2.1.3	aspartate-semialdehyde dehydrogenase	aspartate-semialdehyde dehydrogenase	1.2.1.11	1.2.1.11
P11413	c.2.1.3	glucose-6-phosphate dehydrogenase	glucose-6-phosphate dehydrogenase	1.1.1.49	1.1.1.49
I6XD65	c.33.1.0	nicotinamidase	nicotinamidase	3.5.1.19	3.5.1.19
P0C6D3	c.33.1.0	isochorismatase	isochorismatase	3.3.2.1	3.3.2.1
O42652	c.67.1.0	aspartate transaminase	aromatic-amino-acid transaminase	2.6.1.1	2.6.1.57
Q58696	c.67.1.0	adenosylmethionine–8-amino-7-oxononanoate transaminase	adenosylmethionine–8-amino-7-oxononanoate transaminase	2.6.1.62	2.6.1.62
P07140	c.69.1.1	acetylcholinesterase	acetylcholinesterase	3.1.1.7	3.1.1.7
P19835	c.69.1.1	bile salt-activated lipase	bile salt-activated lipase	3.1.1.13	3.1.1.13
Q9GZT4	c.79.1.0	serine racemase	serine racemase	5.1.1.18	5.1.1.18
Q76EQ0	c.79.1.0	serine racemase	serine racemase	5.1.1.18	5.1.1.18
D2Z027	c.79.1.0	cysteine synthase	cysteine synthase	2.5.1.47	2.5.1.47
P32582	c.79.1.0	cystathionine beta-synthase	cystathionine beta-synthase	4.2.1.22	4.2.1.22
P11954	c.79.1.0	L-serine ammonia-lyase	threonine ammonia-lyase (biosynthetic)	4.3.1.17	4.3.1.19
P9WP55	c.79.1.0	cysteine synthase	cysteine synthase	2.5.1.47	2.5.1.47
Q54HH2	c.79.1.0	serine racemase	serine racemase	5.1.1.18	5.1.1.18
O59791	c.79.1.1	L-serine ammonia-lyase	serine racemase	4.3.1.17	5.1.1.18
O57809	c.79.1.1	1-aminocyclopropane-1-carboxylate deaminase	1-aminocyclopropane-1-carboxylate deaminase	3.5.99.7	3.5.99.7
P47998	c.79.1.1	cysteine synthase	cysteine synthase	2.5.1.47	2.5.1.47
P45040	c.79.1.1	cysteine synthase	cysteine synthase	2.5.1.47	2.5.1.47
P29848	c.79.1.1	cysteine synthase	cysteine synthase	2.5.1.47	2.5.1.47
O70370	d.3.1.1	cathepsin S	cathepsin S	3.4.22.27	3.4.22.27
Q13867	d.3.1.1	bleomycin hydrolase	bleomycin hydrolase	3.4.22.40	3.4.22.40

DRAFT
