# Supplementary material for: Explainable protein function annotation using local structure embeddings
Source: bioRxiv. 2023 Oct 16:2023.10.13.562298. Preprint. [Version 1] doi: 10.1101/2023.10.13.562298 (PMC10614799; doi:10.1101/2023.10.13.562298)
Supplement: Supplementary file 1 [file NIHPP2023.10.13.562298v1-supplement-1.pdf]

bioRxiv preprint doi: https://doi.org/10.1101/2023.10.13.562298; this version posted October 16, 2023. The copyright holder for this preprint (which was not certified by peer review) is the author/funder, who has granted bioRxiv a license to display the preprint in perpetuity. It is made available under aCC-BY 4.0 International license.

Supplementary Materials

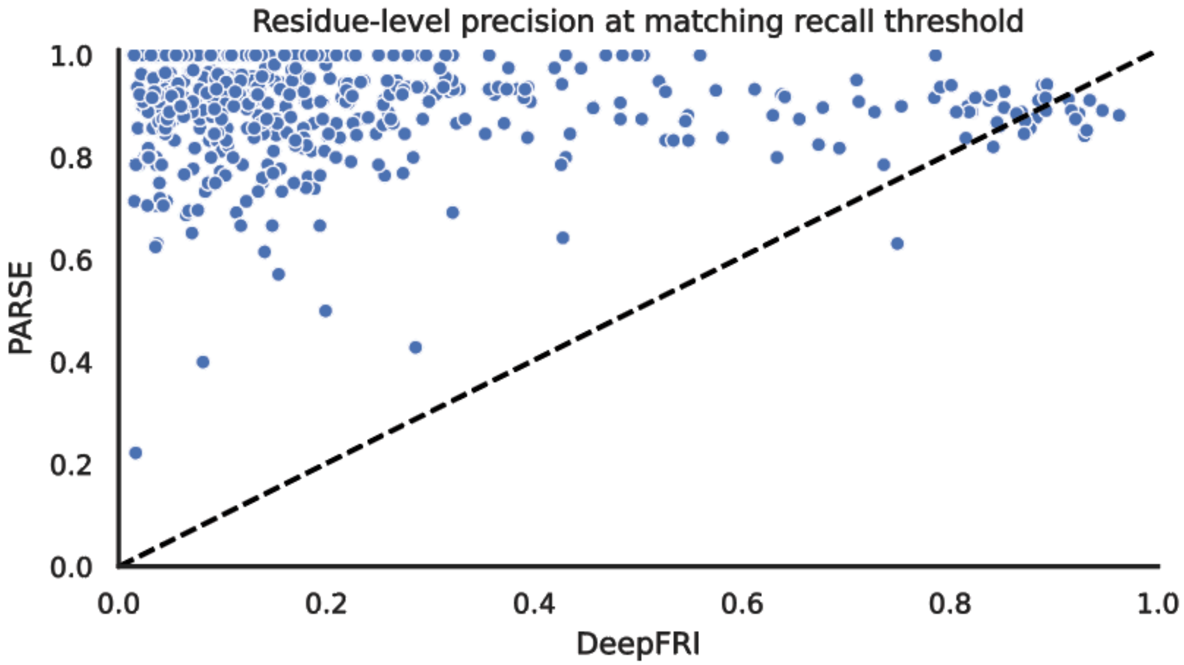

Figure S1. Comparison of residue-level precision for active site prediction. Each dot represents a single protein which was predicted correctly by both PARSE and DeepFRI (n = 599). Precision for DeepFRI was calculated using the threshold corresponding to the recall nearest to the recall achieved by PARSE for that protein.

bioRxiv preprint doi: https://doi.org/10.1101/2023.10.13.562298; this version posted October 16, 2023. The copyright holder for this preprint (which was not certified by peer review) is the author/funder, who has granted bioRxiv a license to display the preprint in perpetuity. It is made available under aCC-BY 4.0 International license.

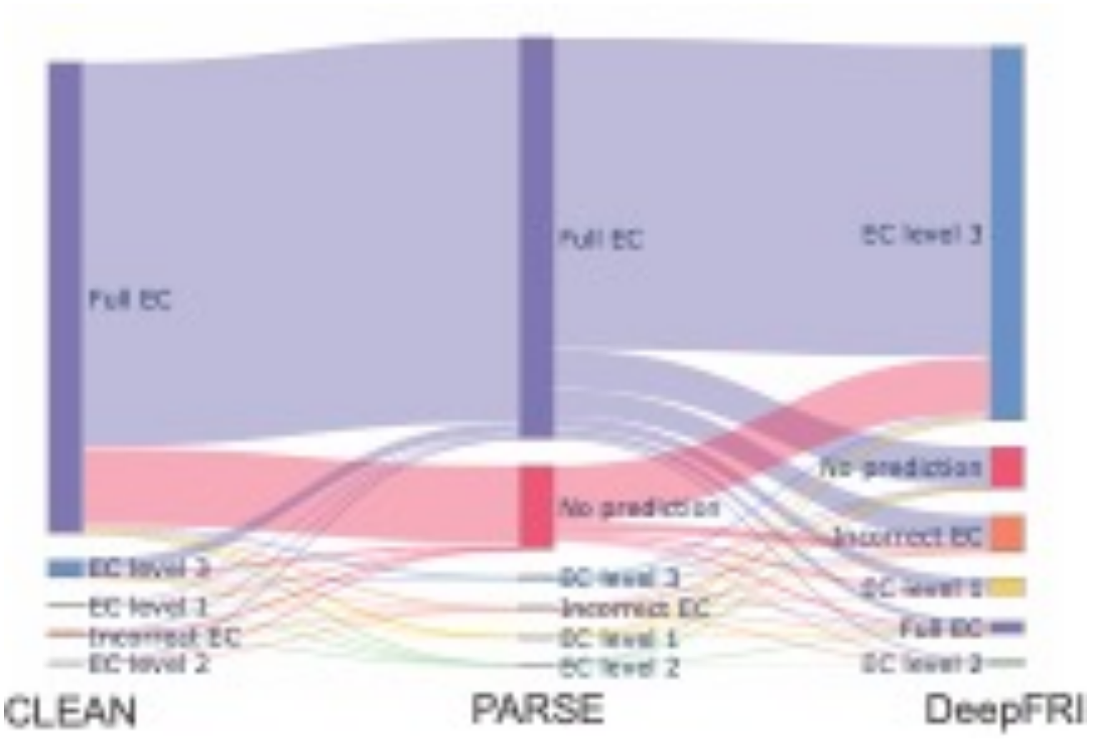

Figure S2. Comparison of top-ranked predictions for each method. Errors are classified into buckets depending on the most specific level of the EC hierarchy they predict correctly (if any).

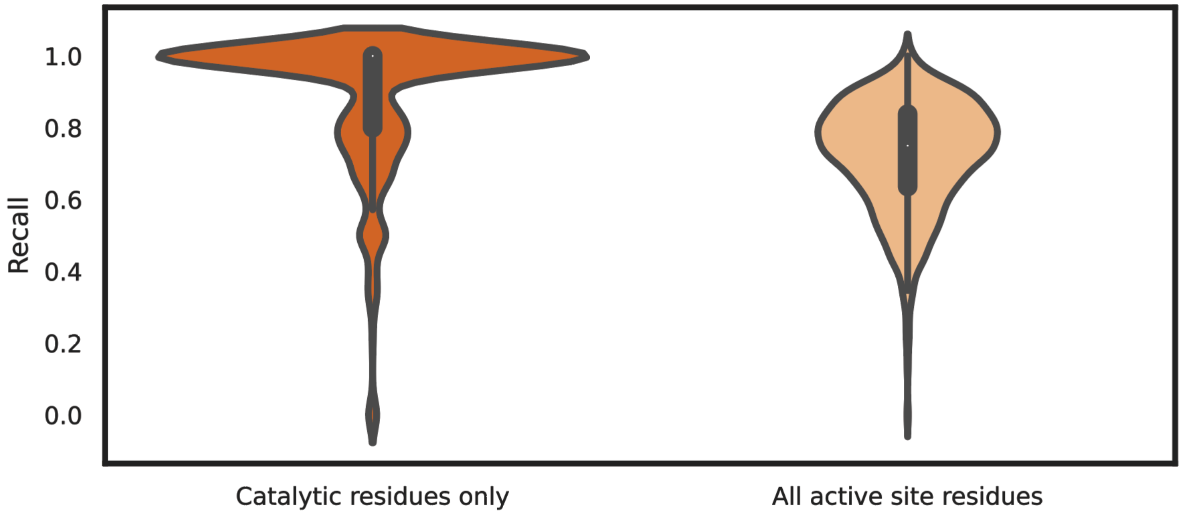

Figure S3. Residue-level recall of PARSE on held-out test set for catalytic residues defined in CSA compared to all residues in the active site (catalytic residues plus neighboring residues within 3.5 Å)

bioRxiv preprint doi: https://doi.org/10.1101/2023.10.13.562298; this version posted October 16, 2023. The copyright holder for this preprint (which was not certified by peer review) is the author/funder, who has granted bioRxiv a license to display the preprint in perpetuity. It is made available under aCC-BY 4.0 International license.

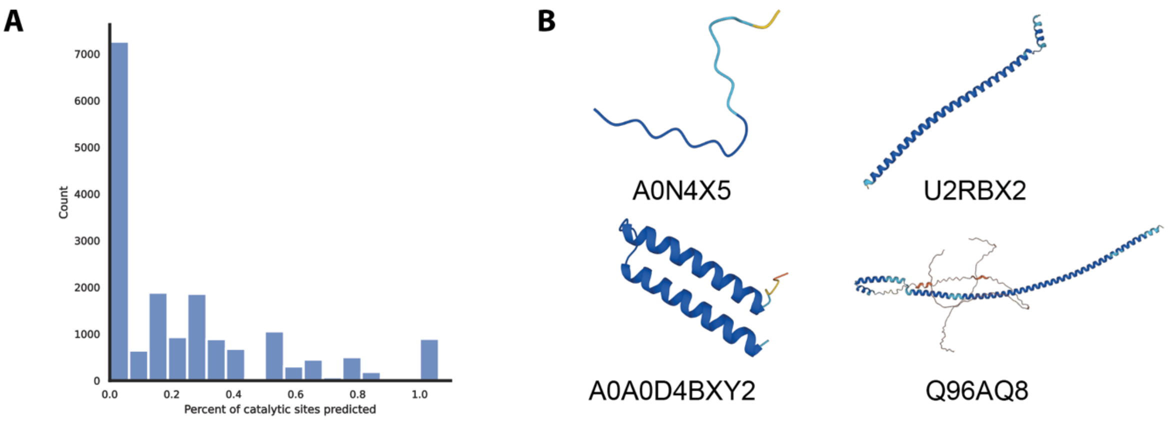

Figure S4. (A) Percent of reference catalytic sites identified for each functional prediction in human proteome. **(B)** Examples of low-complexity predicted structures in the human proteome which have high average pLDDT. Such structures spuriously match many reference proteins, resulting in false positives.

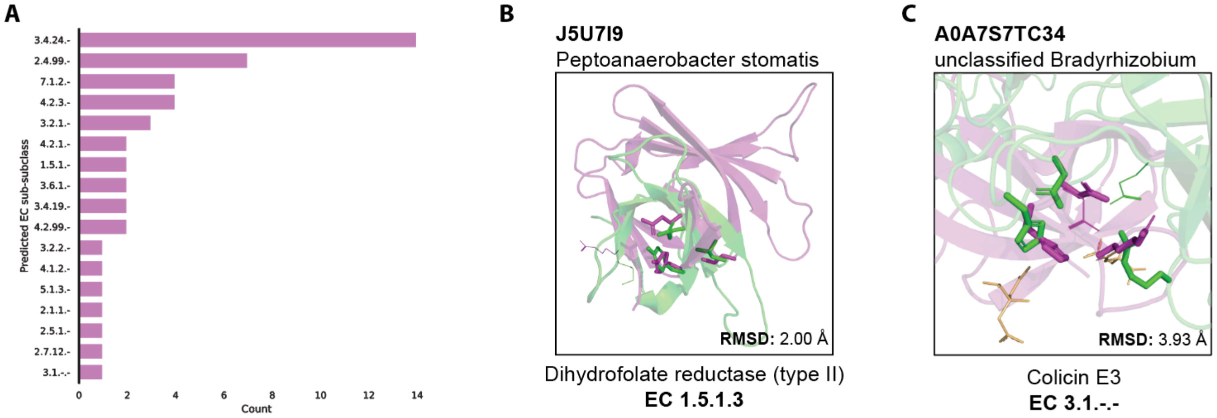

Figure S5. Non-metalloprotease enzymes predicted in the dark proteome. Reference PDB structure is shown in green and the query structure is shown in purple. Catalytic residues from CSA identified by PARSE are shown as sticks, and those that do not match or are not found by PARSE are shown as lines. **(A)** EC number distribution for dark proteome predictions with greater than 75% of reference catalytic residues matching and less than 5 Å RMSD between catalytic residues. **(B)** Example prediction for a DFHR enzyme, with 3 out of 4 catalytic residues matching the CSA reference with low RMSD. The final catalytic residue, Lys32, is replaced by another basic amino acid (Arg) in the same position. **(C)** Example prediction for colicin E3, a ribonucleolytic enzyme. The key Glu-His-Asp catalytic triad is present, albeit in a slightly different orientation, despite highly divergent global folds, suggesting that this protein is an enzyme with a similar mechanism to colicin E3.
